# Introduction to the Toxins Special Issue on Toxicological Effects of Mycotoxin on Target Cells

**DOI:** 10.3390/toxins12070446

**Published:** 2020-07-10

**Authors:** Ana Juan-García

**Affiliations:** Laboratory of Food Chemistry and Toxicology, Faculty of Pharmacy, University of Valencia, E-46100 Valencia, Spain; ana.juan@uv.es

Mycotoxins are toxic secondary metabolites produced by filamentous fungi from *Fusarium*, *Alternaria* and *Penicillium spp*. spread naturally worldwide. Mycotoxins are also natural contaminants present in food and feed and several health problems have been evidenced not only for humans, but also for animals. In 2018, the Rapid Alert System for Food and Feed (RASFF) reported 569 notifications for mycotoxins which were situated among the top 10 food and product hazard categories.

Oral exposure of mycotoxins in humans and animals occurs through food although levels found are low and acute effects are scarce. Nonetheless, chronic exposure effects are of great concern which really pose a significant risk to consumers who are eating these products. Several studies have shown mycotoxins are capable of causing various forms of systemic toxicity such as neurotoxicity, hepatotoxicity, nephrotoxicity, mammalian cytotoxicity, etc. However, these toxicological effects of mycotoxins are evaluated through the extrapolation of results from in vivo and in vitro assays. Studies of mycotoxins’ effects at the cellular level precede those in organs and systems. All these studies are key steps for risk assessment and following legislation for mycotoxins. 

This Special Issue of *Toxins* comprises 10 original contributions and two reviews. The issue reports new findings regarding toxic mechanisms, use of innovative techniques to study the potential toxicity of mycotoxins not only individually but in combination reflecting a real scenario according to nowadays occurrence studies of mycotoxins.

Related with this highlighted scenario, Kifer et al. [1] review the last mathematical models used for evaluating the toxicological effects of mixtures/box/combos/ of mycotoxins. Most of those models are described on the assumption that mycotoxin dose-effect curves are linear; in this review advantages and disadvantages of mathematical models that deal with assessing mycotoxins´ interactions are discussed.

Another review is presented in this Special Issue by Reddy et al. [2], related to less common group of mycotoxins: tremorgenic mycotoxins. Such group of mycotoxins are produced by fungal species often present in association with pasture grasses; indole-diterpenes which may cause toxicity in grazing animals more than in humans directly. Reddy et al., highlight the wide-ranging biological versatility presented by this group of compounds and their role in agricultural and pharmaceutical fields.

Studies of mycotoxins in different cell lines are presented in this issue: intestinal porcine epithelial cells (IPEC-J2), hepatocarcinoma cells (HepG2), mouse macrophage cell line (RAW 264.7), human embryonic kidney cells (HEK 293T), differentiated human intestinal cells (Caco-2 cells), embryonic stem cells (hESCs), human chondrocytes, chicken hepatocytes, wild-type FM3A cells and Bombyx mori (Bm12) cell line.

Transcriptomic analysis based on RNA-seq addressing deoxynivalenol (DON) cytotoxicity in intestinal porcine epithelial cell line (IPEC-J2) showed immune-related pathways to be the main pathway exhibiting more related differentially expressed genes (DEGs). Zhang et al. [3] demonstrated that differentially expressed genes in IPEC-J2 cells treated with DON induced proinflammatory gene expression, including cytokines, chemokines and other inflammation-related genes, by activating p38 and ERK1/2.

Multi-mycotoxin occurrence of 28 mycotoxins in pig feed samples was evaluated by using an LC-MS/MS method. Novak et al. [4] give an overall insight into the amount of fungal secondary metabolites found in pig feed samples compared to their cytotoxic effects in vitro using IPEC-J2 cells. The main mycotoxins, DON and zearalenone (ZEN), were found only at ranks 8 and 10 among all samples analyzed.

Cytotoxicity, genotoxicity and disturbance of cell cycle of two mycotoxins, ochratoxin A (OTA) and beauvericin (BEA), were evaluated in HepG2 cells by Juan-García et al [5]. Effects were evaluated individually and combined through isobologram method and flow cytometry following TG 487 for in vitro micronuclei (MN) assay. BEA showed higher toxicity than OTA in HepG2 cells and combination effects were additive and synergistic. Cell cycle arrest and MN induction were also observed in different scenarios. This study underlines the importance of studying real exposure scenarios of chronic exposure to mycotoxins as mixture of mycotoxins.

Fourteen mycotoxins were individually evaluated in vitro and in different combinations in RAW 264.7 cells, in HepG2 cells and in HEK 293T cells and in silico through ACD/Percepta; the bioavailability potential in differentiated Caco-2 cells is presented (Tran et al. [6]). The mitigation effect with silibinin was also tested. The acute toxicity of these mycotoxins in binary combinations exhibited antagonistic effects in the combinations of T-2 with DON, ENN-A1, or ENN-B, while the rest showed synergistic or additive effects.

OTA is one of the most common and important mycotoxins present in food especially for its potential toxicity. In the work presented by Yang et al. [7], the mechanism underlying the intestinal toxicity of OTA was shown in a dose-dependent manner in differentiated Caco-2 cells. Transcriptome analysis was used to estimate damage to the intestinal barrier; also the protein–protein interaction network by STRING (Search Tool for Retrieval of Interacting Genes) was analyzed, and the validation of eight genes by qRT-PCR analysis was used. Intestinal toxicity of OTA was shown; genome-wide view of biological responses was provided evidencing the theoretical basis for OTA´s enterotoxicity.

A study for evaluating embryotoxicity has been performed by Erceg et al. [8] in embryonic stem cells (hESCs). Results showed mild cytotoxic effects for OTA in these cells by inhibiting cell attachment, survival, and proliferation in a dose-dependent manner.

Two of the most toxic mycotoxins, T-2 and HT-2 toxin, were studied to evaluate apoptosis and autophagy in human chondrocytes through oxidative stress and viability and protein expression (Yu et al. [9]). Decrease of viability was observed for both mycotoxins; while oxidative stress and apoptosis increased but it was higher for T-2 toxin than for HT-2 toxin. The expression levels of apoptosis and autophagy related proteins, Bax, caspase-9, caspase-3, and Beclin1 in chondrocytes induced by T-2 toxin were significantly higher when compared with those levels induced by HT-2 toxin.

Yin et al. [10] in turn studied the toxicological effect of T-2 toxin on apoptosis and autophagy in chicken hepatocytes. At molecular mechanism level, the gene expression level in chicken hepatocytes treated with T-2 toxin induced oxidative stress, apoptosis and cytoprotective autophagy in chicken hepatocytes. This revealed a molecular mechanism of T-2 toxin inducing apoptosis and autophagy in chicken hepatocytes. 

Wild-type FM3A cells and G3 cells (transfected cells with Tri101) were exposed to ITDol, isotrichodermin ITD and trichothecenes (DON, 3-ADON) by Tanaka et al [11]. It was observed that wild-type FM3A cells hardly grew, while G3 cells survived and cytotoxicity was lower in G3 cells exposed to ITDol and ITD than in wild-type FM3A cells. More modest results were detected for in deoxynivalenol and 3-acetyldeoxynivalenol. It indicates that he expression of Tri101 conferred trichothecene resistance in cultured mammalian cells. 

Destruxin A (DA) was studied in the Bombyx mori Bm12 cell line through three proteins (BmTudor-sn, BmPiwi, and BmAGO2) (Wang et al. [12]). Among all three, it was revealed that only BmTudor-sn had an affinity interaction with DA which means there was a susceptibility to DA. When that protein was knocked down, cell viability increased under DA treatment.
